# Profiling metabolic changes in breast cancer with targeted proteomics

**DOI:** 10.1186/2049-3002-2-S1-P7

**Published:** 2014-05-28

**Authors:** Stephan Bernhardt, Devina Mitra, Zita Soons, Rainer König, Martina Vetter, Christoph Thomssen, Eva Kantelhardt, Stefan Wiemann, Ulrike Korf

**Affiliations:** 1 Division Molecular Genome Analysis, German Cancer Research Center (DKFZ), 69120, Heidelberg, Germany; 2 Maastricht University, 6200, Maastricht, The Netherlands; 3 Leibniz Institute for Natural Product Research and Infection Biology - Hans Knöll Institute, 07745, Jena, Germany; 4 University Hospital Halle, 06120, Halle(Saale), Germany

## Background

Breast cancer is one of the most common causes of death from cancer among women. Significant but yet insufficient progress has been achieved due to the introduction of targeted therapeutics [1]. Nevertheless, advancing personalized medicine requires a detailed and comprehensive molecular characterization, particularly at the protein level. This project aims to advance the understanding of crosstalk between cancer-relevant signaling and metabolism.

## Materials and methods

Based on a constructed flux distribution model, targets where chosen to be analyzed in breast cancer cell lines and tumor samples. The RPPA (reverse phase protein microarray) technology a micro-scaled dot-blot approach is used. RPPA allows to quantify protein expression and activation states in a large panel of samples. In addition, RPPA has a remarkable high sensitivity and is able to analyze many proteins in a single experiment. Therefore this method presents a powerful approach for targeted hypothesis-based proteome research [2].

## Results

As a first step, based on publically available transcript data a model describing the deregulation especially of glutamine and nitrogen metabolism in breast cancer was developed [3]. Key features of our model are currently evaluated by RPPA in cell lines representing the different breast cancer subtypes. Metabolic enzymes confirmed on the cell line level as de-regulated will be evaluated in tumor samples and tested as putative drug targets in *in vitro* models.

## Conclusion

Based on the bioinformatic model and proteomic validation our aim is to introduce options for new co-treatment concepts that target key metabolic enzymes to harm tumor cells and disable their malignant anabolic processes, as well as energy consumption. The resulting data will provide a basis to calculate new models explaining the metabolic shift in breast cancer cells and to identify signaling molecules that contribute to malignant transformation.

## References

[B1] Lin SX Chen J Mazumdar M Poirier D Wang C Azzi A Zhou M Molecular therapy of breast cancer: progress and future directions Nat Rev Endocrinol 2010 6 485 493 10.1038/nrendo.2010.92 20644568

[B2] Paweletz CP Charboneau L Bichsel VE Simone NL Chen T Gillespie JW Emmert-Buck MR Roth MJ Petricoin IE Liotta LA Reverse phase protein microarrays which capture disease progression show activation of pro-survival pathways at the cancer invasion front Oncogene 2001 20 1981 1989 10.1038/sj.onc.1204265 11360182

[B3] Curtis C Shah SP Chin SF Turashvili G Rueda OM Dunning MJ Speed D Lynch AG Samarajiwa S Yuan Y The genomic and transcriptomic architecture of 2,000 breast tumours reveals novel subgroups Nature 2012 486 346 352 22522925 10.1038/nature10983 PMC3440846

